# Theoretical Analysis and Characterization of Multi-Islands Single-Electron Devices with Applications

**DOI:** 10.1155/2014/241214

**Published:** 2014-02-05

**Authors:** Amine Touati, Samir Chatbouri, Nabil Sghaier, Adel Kalboussi

**Affiliations:** Faculty of Sciences, Laboratory of Microelectronics and Instrumentation, 5019 Monastir, Tunisia

## Abstract

A two- (2D) and three-dimensional (3D) multiple-tunnel junctions array is investigated. Device structure and electrical characteristics are described. We present a comparison of carriers transport through devices based on polymetallic grains based on master equation and the orthodox theory. The Coulomb blockade effect of 2D and 3D arrays is observed at low and high temperatures. The conduction mechanism is handled by the tunnel effect, and we adopt in addition the thermionic and Fowler-Nordheim emissions. Numerical simulation results focused on flash-memory and photodetector applications. Memory characteristics such as program/erase select gate operation are demonstrated in 2D devices. Also 3D array scheme is discussed for the high-density NCs scalable for photodetector application.

## 1. Introduction

The ITRS [1] is for the last years considering many types of single-electron devices (SEDs) as possible post-CMOS devices in terms of speed, size, power, and some applications. However, the range of applications for SEDs is very large, including sensors, actuators, and optical applications. The quantum effects (discreteness of energy levels) in ultrasmall islands is the important key in such devices. But quantization effects could be problematic for the practical operation; if any dot traps an electron, it blocks other electrons flow due to Coulomb repulsion, and Coulomb blockade (CB) takes place. The technological difficulty is in fabricating SEDs such as single-electron transistors (SETs) [2, 3] in the nanometer size range. An alternative approach is the multi-islands SETs; multi-island devices can be considered as multiple SETs connected to each other in series or in parallel; one of the major advantages of multi-island device comes from the fact that it requires a much simpler fabrication procedure than single-dot structures. Several multi-island SETs based on poly-Si [4], metal [5, 6], and Si quantum dots (QD) [7] have already been reported. In particular, metal nanocrystal (NC) was proposed above its semiconductor counterpart due to the selectable work function and large density of states [8, 9]. However, theoretical models of one-dimensional array NC memories have been investigated [10]. Therefore, analyzing the exact theory of 2D and 3D arrays is a very complex task, so we used the MC simulator.

This work will show the advantage of MTJs in the context of a comparison of various SETs with MTJs structures (2D and 3D) and report the specific electrical properties of these devices, operated from 4.2 K to 300 K. In the other hand we investigate two applications.

## 2. Device Structure and Parameters

**Figure 1:**
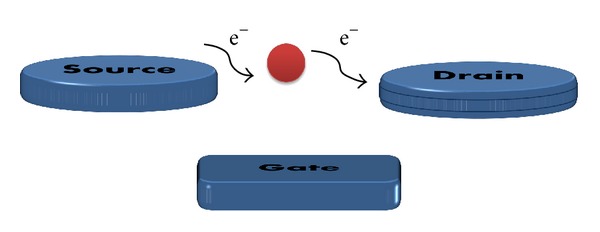
Basic structure of SETs schematics.

Single electron transistors (SETs) architecture in Figure 1 is quite similar to the MOSFET architecture, with a source, a drain, and a gate. The main difference is that, in the SETs configuration, the channel is replaced by an ultrasmall conductive island (capacitively coupled to the gate, where a gate capacitance *C*_*G*_) and separated from source drain by two tunnel barriers. The tunnel junctions are electrically defined by a tunnel capacitance (*C*_*T*_) and resistance (*R*_*T*_). The operation of SETs exploits the discrete number of charges in the conductive island. The gate voltage *V*_*G*_ may be used to control the Fermi level of the island and overcome or impose a Coulomb blockade. When the voltage *V*_*G*_ lowers the island Fermi energy, the energy difference between the source and island caused by the single-electron charging energy is overcome. Electrons can then transfer from source to drain, across the island and a current is observed.

**Figure 2:**
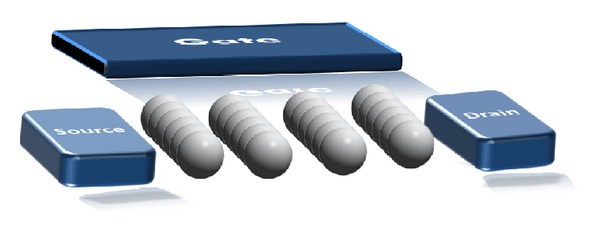
The 4 × 6 regular multi-islands systems. Each island is controlled electrostatically by the gate and is separated from its neighbor by a tunnel junction.

It is also possible to observe single-electron charging effects with more than one island, connected to each other and to the electrodes by tunnel junctions. Such a system is referred to as a multiple-tunnel junction (MTJ). The equivalent circuits of the two-dimensional (2D) of multi-islands SETs used for simulations are shown in Figure 2. For the simulations, the grains are electrically modeled as dots connected to their neighbors by tunnel junction, capacitances *C*_*T*_, tunnel resistances *R*_*T*_, and sharing a common gate. A gate is coupled by a capacitance *C*_*G*_, to each dot. Also a second gate can be added to the device.

**Figure 3:**
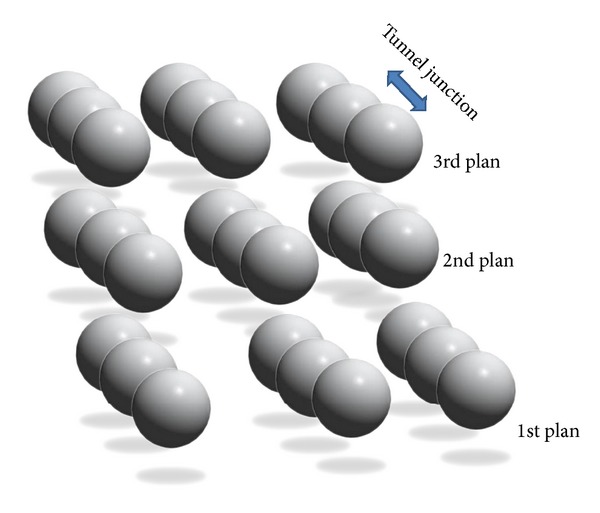
Three planes of 3 × 3 × 3 regular multi-islands structures.

The other proposed type of multi-islands SETs is the three-dimensional (3D) structure. In this case we have arranged the 2D structure in a vertical succession. In Figure 3 we have schematically a structure of three plans of 3 × 3 × 3 regular dimensional MTJs. This system may have one or two or gates although a source-drain voltage (*V*_DS_) for each bloc.

## 3. Results and Discussions

### 3.1. Monte Carlo Simulation of Structures Characterizations at Low and High Temperatures

In order to calculate the *I*-*V* curves in the device, taking into account both the temperature and the through junctions, was simulated using the standard Monte Carlo (MC) simulations (using SIMON software [11]) which appear to be an adequate tool for the prediction of electrical behavior of multiple-dot systems connected in arrays of tunnel junctions. The most general description of single electron tunneling in the model used in SIMON is to solve the master equation for the occupation probabilities of each state:

(1) $$\begin{array}{c} \frac{\partial P_{i}\left(t\right)}{\partial t}=\underset{j\neq i}{\sum }\left[\Gamma_{ij}P_{j}\left(t\right)-\Gamma_{ji}P_{i}\left(t\right)\right]. \end{array}$$

*P*_*i*_(*t*) is the time-dependent probability that where device has charge *i*, and Γ_*ij*_ is the tunneling rate from state *i* to state *j*.

However, grain sizes, tunnel capacitances, and tunnel resistances have to be very well known or estimated. For the simulations in 2D, the dots are electrically modeled as dots connected to their neighbors by four tunnel junctions with tunnel capacitances of 0.1 aF and tunnel junction resistances of 2 MΩ. The gate capacitance is about 0.01 aF and is connected with the first plan of dots. These values are used by estimations derived from geometrical assumptions based on [12]. For the 3D the dots are connected to their neighbors by six tunnel junctions; the tunnel capacitances and resistances take the same values of 2D. Consequently, each NC can be considered as QDs and these QDs may behave as excellent traps for one or few electrons (resp., photon).

**Figure 4:**
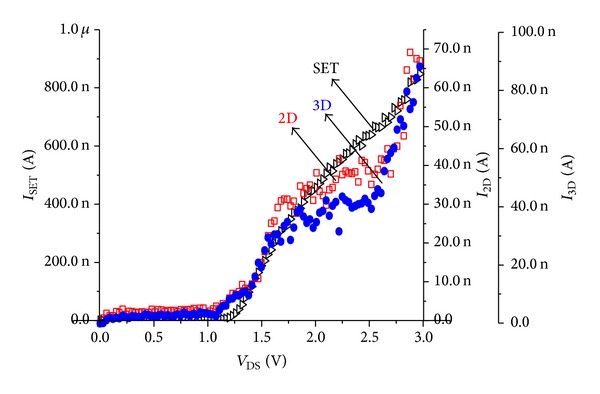
*I*-*V*_DS_ characteristics of a SET and 2D and 3D at 300 K.

Figure 4 shows MC simulated drain current versus drain voltage (*I*-*V*_DS_) characteristic for a simple SET, 2D (3 × 5 dot arrays) and 3D (3 × 3 × 5 dots arrays) devices at 300 K. Although the tunnel junction capacitances and gate capacitances in a single-dot SET are much lower than the intrinsic capacitance of a high temperature operation (*C*_Σ_ ≪ *e* ^2^/2*k*_*B*_ *T* where *C*_Σ_ is the island capacitance, *e* is the elementary charge, *k*_*B*_ is the Boltzmann constant, and *T* is the temperature of system), the behavior of the SET loses its basic property: Coulomb gap (*C*_*G*_). However, we consider that CB phenomenon can be observed only in SETs with asymmetric structures. On the other side, the curves are nonlinear with a current; the transistor effect clearly persists in the 2D and 3D dot arrays: open/closed window. The nonlinear behavior is more pronounced at room temperatures, which is a sign of the Coulomb blockade of electron tunneling. However, this behavior alone cannot prove the existence of a charging effect; each island gives an extra Coulomb blockade region. We note that the total island capacitance *C*_dot_ in the 2D structure per array becomes (4*C*_*T*_ + *C*_*G*_)/2, and for the 3D structure it is (5*C*_*T*_ + *C*_*G*_)/3 for the upper and under planes and 2*C*_*T*_ for the planes between them due to the series and parallel combination of *C*_*T*_. For *V*_DS_ lower than ~1.1 V, the multi-islands device is electrically blocked. Tunnel conduction takes place when *V*_*D*_ is between 1.1 V and 1.5 V. For *V*_DS_ higher than 1.5 V, the Fermi-level energy of electrons is decreased and the island energy level becomes transparent to it; thermionic conduction takes place. Yet, the current decreased when the number of NCs increased; this is observed experimentally [4]. Compared to a simple SET the drain polarization is higher; this is relative to the energy level of the big system of NCs arrays. Consequently, the arrays system can be considered as multi-SETs connected and successively turn “on” and “off,” for transferring small packet of electrons; then width of CB region now is multiplied by the number of SETs per array, so the voltage is applied. The electron passes from a junction to other, repeating this process for successive islands, and for islands lying after the *k*th island, it gives us the potential of the *ℓ*th island. The potential according to the law [13] is

(2) $$\begin{array}{c} \phi_{\ell }=-\frac{e}{C_{\text{dot}}}e^{-\left|k-\ell \right|/M},\;\text{where}\;M={\left(\ln\left(\frac{C_{\text{dot}}+C_{g}}{C_{\text{dot}}-C_{g}}\right)\right)}^{-1}. \end{array}$$

**Figure 5:**
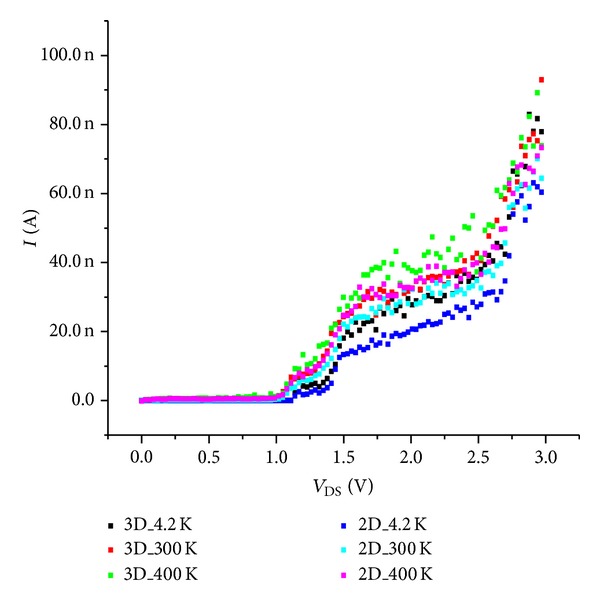
Temperature effect on the 2D and 3D source-drain currents.

Figure 5 compares the characteristics of two types of equivalent MTJs arrays devices at different temperatures. This characteristic shows that low and high temperatures have no remarkable effect on the functioning of the system, but we note that the thermionic effect has more impact on 3D at high temperature (400 K) than on 2D arrays as a result of three-dimensional current density.

A Coulomb gap of 1 V is seen at *V*_*G*_ = 0 V for 4.2 K and 300 K. This proves that the tunnel current persists for the low source-drain voltage (~1.3 V) and thermionic current for the high polarization. Therefore, replacing the single island by multiple islands, multi-island suppresses the cotunneling effect.

**Figure 6:**
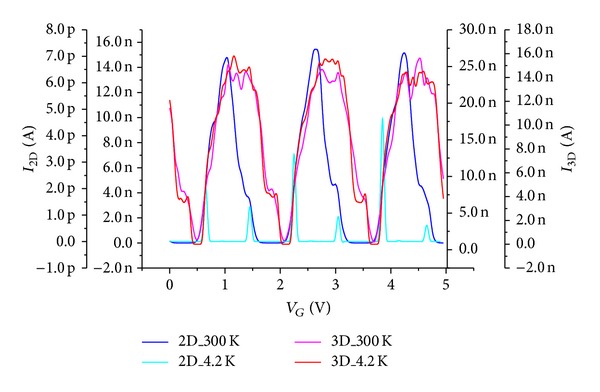
Monte Carlo simulation of Coulomb oscillation for 2D and 3D structures at low and high temperatures for *V*_DS_ = 0.5 V.

Figure 6 demonstrated, at low and room temperatures, that, for such structure, the number of oscillations is maximal at 4.2 K for 2D structure, and an extra valley of Coulomb oscillation for the 3D device at range of temperatures (4.2 K and 300 K) same the SET device. Single-electron current oscillations in the *I*_*D*_-*V*_*G*_ characteristics with a period Δ*V*_*G*_ are 0.8 V at 4.2 K and 1.6 V at 300 K for 2D device and for the 3D device are 1.55 V at 4.2 K and 1.7 V at 300 K. The oscillations correspond to the addition of electrons one by one to a dominant charging grain. The periodic Coulomb oscillations correspond to homogeneous one-dimensional array system with the same size. Trapping centers appeared in the 2D structure, while in the 3D structure we see well that the excitement of these centers is possible.

**Figure 7:**
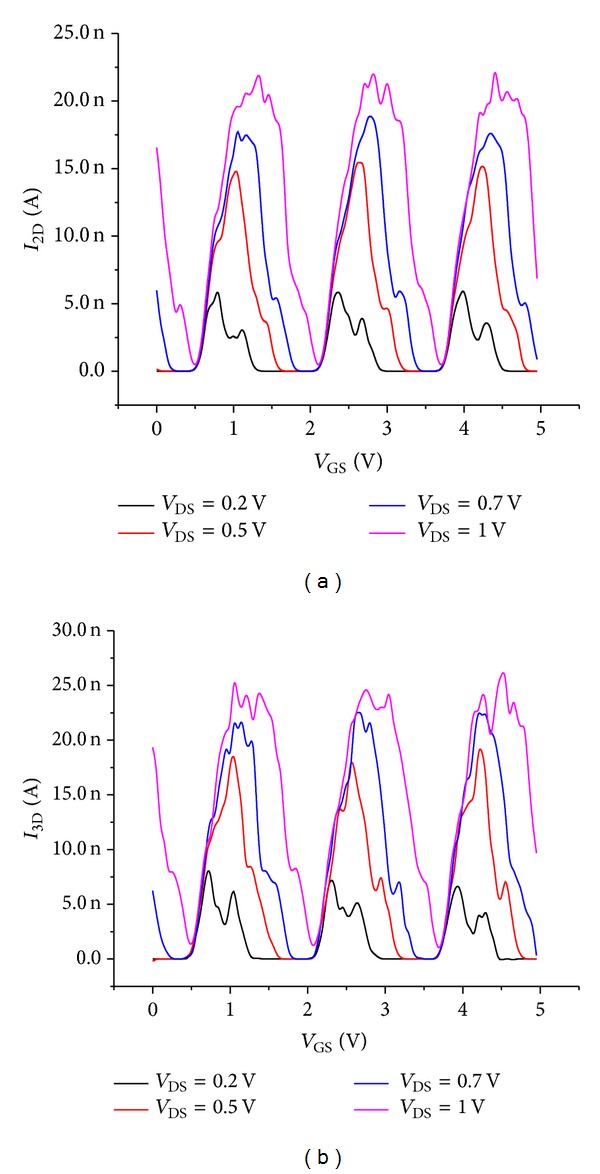
Gate current simulation at 300 K for different drain voltages, (a) *I*_2D_-*V*_GS_ and (b) *I*_3D_-*V*_GS_.

Therefore, as the gate voltage is increased at constant drain voltage, more electrons are flowing through the array and thus the gate current increases. The peak number and intervals of CB did not change in the temperature range from 4.2 K to 300 K. The tunneling current increases with increasing temperature and oscillations are becoming much more clearly periodic. It is interesting to note that increasing the temperature could allow a recovery of oscillations periodicity similar to the SETs. As the temperature increases the thermal energy *k*_*B*_ *T* (0.025 eV at 300 K) becomes lower than 2*e* ^2^/(4*C*_*T*_ + *C*_*G*_) in 2D (0.78 eV) and 3*e* ^2^/(5*C*_*T*_ +  *C*_*G*_) in the 3D arrays (0.91 eV); this is shown in Figure 6 by small widths of CB zone in the 2D than the 3D devices at room temperature and large widths of CB at low temperature. Simulation of *I*-*V*_*G*_ represented at 300 K for four different drain voltages shows that the Coulomb oscillation should increase with an increase of the drain voltage in the 2D and 3D arrays. This is verified on Figure 7 where the high regularity of resulting oscillations makes their exploitation easy for nanoelectronics integrated circuit applications. On the other hand, to achieve a sufficient memory window, a dense NC array is favorable while taking the trade off with the NC number into account.

### 3.2. 2D Devices Flash Memory

For the breakthrough of the scaling limitations [14], 3D stacked memory arrays are under development [15, 16]. At the beginning, NAND flash memories with just stacked structures of the conventional planar-type device with single NC or poly-NCs channels were considered [17, 18]. Then, they have been advanced to reduce the process cost further with unique 2D array architecture of metallic NCs [19, 20].

We have shown that the MTJs arrays can be used as a current sensing device, very low current measurements, for high voltage bias. The NC memory remains among the most promising because of its compatibility to the current. Multi-island devices may be more suitable for memory applications because the size dispersion and position of the islands are less restricting [21]. In the flash-memory array [22], each intersection of bit and word lines becomes a memory node.

**Figure 8:**
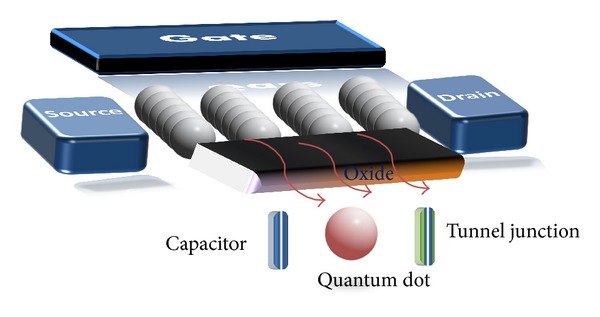
Nonvolatile flash-memory cell proposed with 2D structure.

However, in the word-line stacked memory array, it is difficult to form single-crystal channels because the bit lines are formed after stacking multiple word lines and dielectrics. As a result, performances are degraded and a large dispersion of electrical characteristics among cells is resulted. In Figure 8 we schematize the memory cell; it consists of 2D MTJs arrays coupled with a single-electron box (SEB) [23, 24]. The coupling is with an oxide modeled by a capacity *C*_*C*_. “Set” and “Reset” states can be done within a single step from *V*_GS_ or *V*_DS_.

Initially the memory cell stores the logical “0,” that is, no excess electrons are present in the memory node. For writing “1,” a positive bias is first applied, which corresponds to 1 electron in the memory node. A negative voltage pulse is then applied to inject electron outside the memory node.

**Figure 9:**
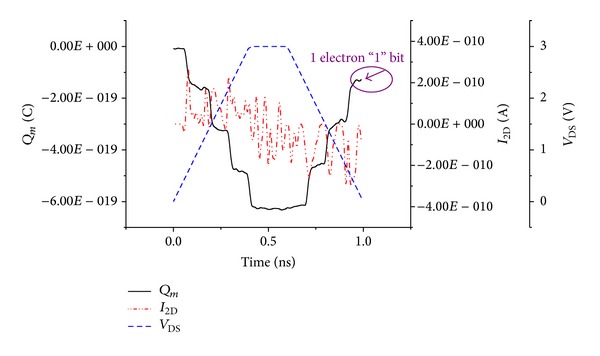
Simulation of the logical “1” at 300 K. The applied write voltages induce a 2D-MTJs oscillations current for traped electron.

To add one electron to the memory dot requires a voltage increment of *e*/*C*_2D_ to be applied to the memory voltage, where *C*_2D_ is the total capacitance 2D system.  Figure 9 shows the memory operation characteristics on the node memory of SEB, where four electrons at 300 K are confined on the QD memory when the *V*_DS_ takes the value of 3 V. Further the polarization is swept from 3 V to 0 V, 3 electrons are ejected from the QD memory, and a hysteresis current appears in the electron accumulation regime of the 2D MTJs. This hysteresis can be explained by reading the bit “1” with a current drop when the 3 electrons are ejected. So, the electrostatic [25] and quantum confined phenomena are the most key effects of this memory effect. Note that current hysteresis is reproducible for various bias voltages. This is attributed to charges stored in nearby, additional QD attached on the gate electrode. The program/erase speed of the memory cell structure shows fast transient characteristics, even at the low program/erase bias conditions due to the field-concentration effect.

**Figure 10:**
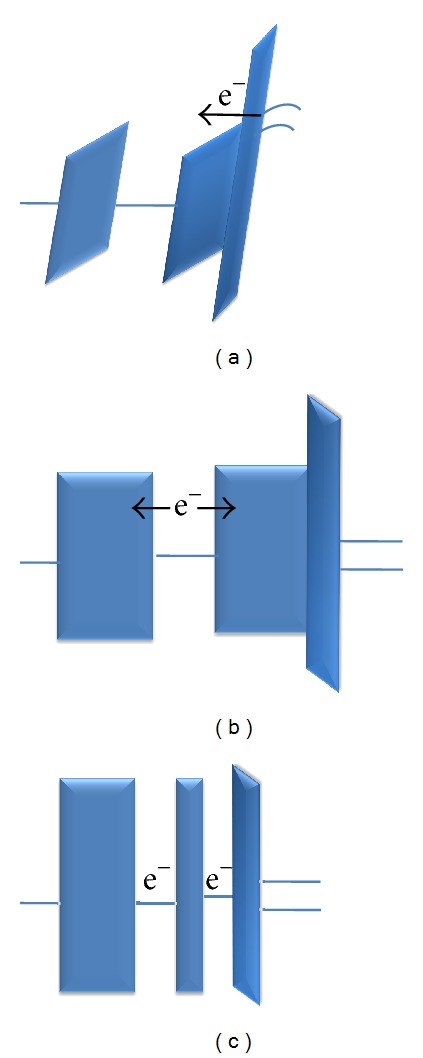
Energy band diagrams of a conventional structure (a) during programming and (b) during retention status and (c) multinanocrystals retention.

The concept of tunneling is dependent on the memory status of energy barrier. Generally, the barrier height *φ* of conventional tunnel junction is not influenced by the gate voltage, regardless of whether the system is in program/erase status or in retention status, as shown in Figure 10.

### 3.3. 3D Devices Photodetector

**Figure 11:**
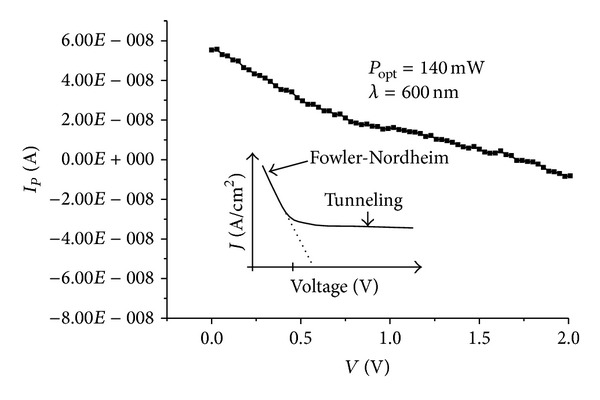
Photogenerated current characteristics of a 3D array under illumination pulse with *λ* = 600 nm at room temperature.

In this part we propose a new 3D photodetector based on a multi-NCs array for a single charge detection (photo-3D SET) model. A recent model was proposed for photo-SET (single electron photodetector) aiming at detecting one by one electron [26, 27]; the device structure presented is consisting of two SETs capacitively coupled. The presented device structure consists of one bloc (reading and detection blocs) that operates simultaneously. The upper part of the 3D structure is illuminated by light. The source of light applied on the 3D MTJs is modeled as a power source delivering a current *I*_*P*_ = *I*_0_ × cos⁡ (2*πft*). The number of electrons in QDs assigned the conductance of second arrays below. When a photon of an energy quantum *E* = *hν* is absorbed by any QDs, an excited electron with the *E* energy excess will rapidly escape. The photogenerated current *I*_*p*_ is proportional to the incident optical power *P*_opt_ and is given by

(3) $$\begin{array}{c} I_{p}=R_{d}P_{\text{opt}}=\frac{P_{\text{opt}}\eta q}{h\nu }=\frac{P_{\text{opt}}\eta \lambda \;\left(\mu \text{m}\right)}{1.24}, \end{array}$$

where *R*_*d*_ is called the responsivity of the photodetector, *η* is the quantum efficiency, *hν* = *hc*/*λ* is the photon's energy *h* is Planck's constant, *ν* is velocity of light, and *q* is the electronic charge. Light scattering and absorption enhancement were experimentally observed in vertical metal-oxide NW arrays [28]. In our case we have metal NCs coupled with tunnel junction, aligned along a perpendicular direction, and dispersed horizontally; upon illumination the electrical conductivity increases at photon energies being photogenerated; in Figure 11 we present the photocurrent characteristics *I*_*p*_ versus pulse voltage *V* for one second. The origin of this observation is attributed to an increase in NCs diameter under illumination due to local heating and electrons are photoexcited from the dots to the near continuum states. The electrons may be captured by successive dots in transit. Under illumination, photoemission from the QDs contributes to an additional current component, the photocurrent, and an additional trapping into the quantum dots. The physical mechanism responsible for photoconductive gain in QDs is Fowler-Nordheim type. Under no illumination, the current through the device is due to generation-recombination processes resulting from carrier trapping into and thermionic emission from the quantum dots.

## 4. Conclusions

We have given a broad overview on different multi-island SETs, in two-dimensional and three-dimensional, and their possible applications. MC simulations performed prove the regular CB phenomena in 2D and 3D systems at low temperature and at room temperature with high voltage bias; make their exploitation for SET applications. To clarify the advantage from the viewpoint of operating temperature the total capacitance of the multiple island system was decreased than a SET with a single island, since the operation temperature becomes three times higher by multiarray islands. Memory application is also demonstrate with 2D the electrostatic and quantum confined is the memory effect of stored bit. We have shown the possibility to develop novel photodetector concepts based on three-dimensional array effects with metallic quantum dots.
